# Prevalence of Severe Mental Illness and Its Associations With Health Outcomes in Patients With CKD: A Swedish Nationwide Study

**DOI:** 10.1053/j.ajkd.2024.12.004

**Published:** 2025-02-25

**Authors:** Nanbo Zhu, Anne-Laure Faucon, Ralf Kuja-Halkola, Mikael Landén, Hong Xu, Juan Jesús Carrero, Marie Evans, Zheng Chang

**Affiliations:** Department of Medical Epidemiology and Biostatistics (NZ, A-LF, RK-H, ML, JJC, ZC), Division of Clinical Geriatrics, Department of Neurobiology, Care Sciences and Society (HX), and Department of Clinical Science, Intervention and Technology (ME), Karolinska Institutet, Stockholm, and Department of Psychiatry and Neurochemistry, Institute of Neuroscience and Physiology, Sahlgrenska Academy, University of Gothenburg, Gothenburg (ML), Sweden; and Department of Clinical Epidemiology, Centre for Research in Epidemiology and Population Health, INSERM U1018, Paris-Saclay University, Villejuif, France (A-LF).

## Abstract

**Rationale & Objective:**

Patients with chronic kidney disease (CKD) often face mental health problems, but the burden of severe mental illness (SMI) in this population is unclear. We estimated the prevalence of SMIs among people with CKD and their associations with health outcomes.

**Study Design:**

Nationwide cross-sectional and cohort study.

**Setting & Participants:**

Using the Swedish Renal Registry, we identified 32,943 patients with incident CKD G3b-5 or kidney replacement therapy (KRT) between 2008 and 2020 for estimation of the prevalence of SMIs. Data about the 30,103 patients not receiving KRT were used to examine associations between SMIs and subsequent health outcomes.

**Exposure:**

Occurrence of SMIs (ie, schizophrenia, bipolar disorder, and major depressive disorder) before the date of first registration into the registry (index date), using diagnoses from inpatient or specialist outpatient care.

**Outcome:**

30% decline in eGFR, initiation of KRT, and all-cause mortality.

**Analytical Approach:**

Prevalence of SMIs was estimated in patients with CKD and compared with the general population using standardization with ratios adjusted for age, sex, and calendar year. Associations between SMIs and health outcomes were examined using Cox proportional hazards models.

**Results:**

The overall prevalence of SMI was 7.3% in patients with CKD, which was 56% higher than the general population. The prevalences for schizophrenia, bipolar disorder, and major depressive disorder were 0.5%, 2.1%, and 5.6%, respectively. All 3 SMIs were associated with a higher mortality rate. Schizophrenia was not associated with 30% decline in eGFR (HR, 0.92 [95% CI, 0.65–1.29]), but it was associated with a lower rate of initiating KRT (HR, 0.56 [95% CI, 0.39–0.80]). Bipolar disorder was associated with a higher rate of 30% decline in eGFR (HR, 1.47 [95% CI, 1.29–1.67]) but a lower rate of initiating KRT (HR, 0.79 [95% CI, 0.67–0.94]). Major depressive disorder was not associated with 30% decline in eGFR or initiation of KRT.

**Limitations:**

Lack of primary care data and exclusion of individuals with CKD G1–3a.

**Conclusions:**

Patients with CKD had a higher prevalence of SMI compared with the general population. In patients with CKD, each SMI was associated with higher mortality, and bipolar disorder was associated with a faster eGFR decline. Patients with CKD and pre-existing schizophrenia or bipolar disorder experienced a lower rate of initiating KRT.

## PLAIN-LANGUAGE SUMMARY

Patients with chronic kidney disease (CKD) frequently experience mental health problems, yet the prevalence and impact of severe mental illness (SMI) in this population remain uncertain. This Swedish nationwide study revealed that the prevalence of any SMI was 7.3% among patients with CKD (0.5% for schizophrenia, 2.1% for bipolar disorder, and 5.6% for major depressive disorder), representing a 56% higher prevalence than experienced by the Swedish general population. All 3 SMIs were associated with a higher mortality rate in patients with CKD, and bipolar disorder was also associated with a faster eGFR decline. Moreover, patients with CKD and schizophrenia or bipolar disorder exhibited a lower rate of initiating kidney replacement therapy. These findings highlight the need for improved recognition and management of SMI among people with kidney disease.

## Graphical Abstract

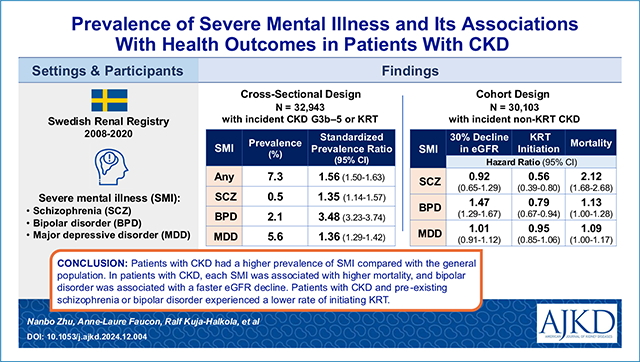

Over 10% of adults worldwide are affected by chronic kidney disease (CKD), which encompasses a spectrum of disease severity from mild decline to near-total loss of kidney function.^1^ When progressing into kidney failure, kidney replacement therapy (KRT) including maintenance dialysis and kidney transplantation is the essential life-sustaining treatment option, yet is subject to limited availability and disparate accessibility.^2^ Patients with CKD often experience psychological distress and neuropsychiatric symptoms; the prevalence of major depressive disorder and anxiety disorders is doubled or tripled compared with the general population,^3,4^ and cognitive impairment is remarkably common.^5^ However, there is a notable lack of knowledge about schizophrenia and bipolar disorder, less common but serious conditions, in the CKD population.^6,7^ Collectively, schizophrenia, bipolar disorder, and major depressive disorder are often referred to as severe mental illness (SMI) due to their substantial negative impact on quality of life, physical health, and life expectancy.^8–10^

The importance of managing mental illness in patients with CKD has received increasing attention.^11,12^ The co-occurrence of CKD and SMI could be partially explained by established risk factors, such as unhealthy lifestyles, cardiometabolic diseases, and psychotropic medication use (eg, lithium).^6,7^ A meta-analysis demonstrated that about 20% of nondialysis CKD and dialysis patients had a major depressive disorder,^3^ and the few available studies in nondialysis CKD patients indicated a prevalence of 1.2% for schizophrenia^13^ and 1.5% for schizophrenia/bipolar disorder.^14^ Nevertheless, these studies lacked a refined disease definition (eg, additional inclusion of other psychotic disorders), complete coverage of inpatient and outpatient data, or representativeness of the entire population. There is a need to better characterize the prevalence of SMI across the spectrum of CKD.^6^

Patients with SMI often encounter significant adverse health outcomes as well as disparities in health care access.^9,15^ Previous studies have found depression/depressive symptoms to be associated with elevated rates of hospitalization, CKD progression, cardiovascular disease, and all-cause mortality.^16–19^ Limited research in the CKD population has suggested that schizophrenia or bipolar disorder are associated with higher rates of hospitalization and mortality.^20–23^ However, these studies have important limitations, including the absence of data on nondialysis CKD patients,^21,22^ lack of investigation into kidney function decline,^20–23^ reliance on inpatient diagnoses of SMI,^21–23^ or grouping SMI diagnoses together.^21,23^ Understanding the burden of SMI in patients with CKD remains a priority to provide appropriate support and improve health outcomes for individuals with CKD and coexisting SMI.^6,7^

In this Swedish nationwide study, we (1) estimated the prevalence of SMIs (ie, schizophrenia, bipolar disorder, and major depressive disorder) in patients with CKD, and (2) examined how SMIs influence kidney-related outcomes and all-cause mortality.

## Methods

### Data Source

We used data from the Swedish Renal Registry, a nationwide quality register that collects longitudinal information on patients attending outpatient nephrology care with nondialysis CKD or undergoing KRT since 2008.^24,25^ The register’s primary enrollment criterion is an eGFR < 30 mL/min/1.73 m^2^ (ie, CKD stages G4–5) during nephrology visits, but it also encourages the inclusion of patients at an earlier stage, mainly those with an eGFR < 45 mL/min/1.73 m^2^.

Almost all nephrology clinics in Sweden report to the Swedish Renal Registry, with estimated national coverage of >75% for patients with CKD G4–5 and >95% for patients on dialysis.^24,25^ All patients were informed about their participation in this register and could refuse participation or withdraw registration anytime. The Swedish Renal Registry was used to identify the CKD population and ascertain kidney-related outcomes. It was linked to several national registers using each individual’s unique personal identification number, enabling the acquisition of detailed health-related data, such as clinical diagnoses, dispensed medications, and vital status.

The National Patient Register provided clinical diagnoses coded according to the *International Classification of Diseases, Tenth Revision* (ICD-10) from hospital admissions since 1997 and from outpatient specialist consultations since 2001.^26^ The Cause of Death Register provided information on the dates and underlying causes of all deaths in Sweden.^27^ The Prescribed Drug Register provided complete information on all prescribed drugs dispensed at Swedish pharmacies since 2005.^28^ A graphical depiction of data coverage and measurement is presented in Figure S1. In addition, the Swedish Total Population Register served as a source for general population controls.^29^ Through a separate linkage of the Total Population Register and the National Patient Register, we estimated the prevalence of SMI in the general population.

The study was approved by the Swedish Ethical Review Authority (project number: 2018/1591-31/2, 2022-04594-02). Informed consent is not required for pseudo-anonymized register-based research according to Swedish law.

### Study Design and Sample

**Figure 1.**
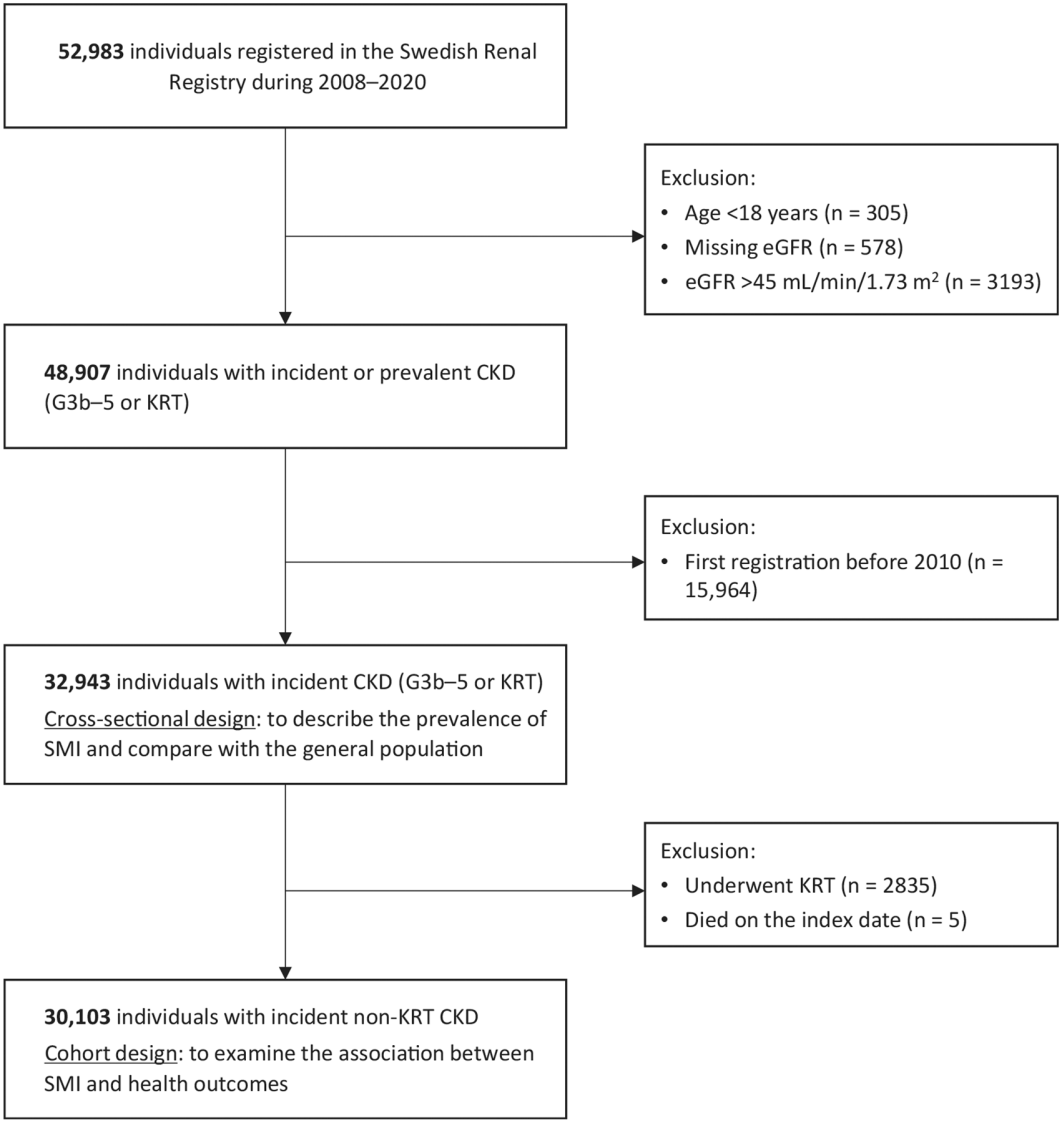
Flowchart of the sample selection. Abbreviations: CKD, chronic kidney disease; eGFR, estimated glomerular filtration rate; KRT, kidney replacement therapy; SMI, severe mental illness.

We employed 2 study designs to address 2 aims. First, a cross-sectional design was used to estimate the prevalence of SMI in patients with incident CKD and compare it with the prevalence in the general population. We included all adults (≥18 years) with an eGFR < 45 mL/min/1.73 m^2^ or undergoing KRT who were registered in the Swedish Renal Registry between 2008 and 2020. The index date was defined as the first registration date during the study period. We calculated eGFR using the 2009 CKD Epidemiology Collaboration equation without the race coefficient,^30^ based on serum or plasma creatinine tests traceable to isotope dilution mass spectrometry standards. Stages of CKD were determined by baseline eGFR level and KRT status, categorized into G3b (eGFR 30–44 mL/min/1.73 m^2^), G4 (eGFR 15–29 mL/min/1.73 m^2^), G5 (eGFR < 15 mL/min/1.73 m^2^ not receiving KRT), and KRT.^31^ Since the Swedish Renal Registry attained national coverage in 2008 and initially included prevalent CKD patients, we identified patients with incident CKD G3b-5 or KRT by excluding individuals registered before 2010 (Fig 1).

Second, a cohort design was used to examine the associations of SMI with subsequent health outcomes. The study cohort was restricted to patients with incident non-KRT CKD because KRT was considered an outcome of interest.

### Severe Mental Illness

Occurrence of SMI before the index date, including schizophrenia (ICD-10 code F20), bipolar disorder (F30-F31), and major depressive disorder (F32-F33), was ascertained from the National Patient Register. Schizophrenia spectrum disorders (F20-F29) were used as a secondary exposure. Previous research has demonstrated good validity of the ICD diagnoses of schizophrenia spectrum disorders (positive predictive value, 0.94) and bipolar disorder (0.92) in the Swedish National Patient Register.^32,33^ There are currently no validation studies available for major depressive disorder.

### Health Outcomes

Primary outcomes included a sustained >30% decline in eGFR, initiation of KRT, and all-cause mortality. A 30% eGFR decline is frequently used as a surrogate end point for kidney disease progression.^34^ We estimated the eGFR slope with all available eGFR measurements during the non-KRT follow-up period using a linear mixed model.^35^ To be considered a “sustained” eGFR decline, the eGFR slope for an individual needed to be negative, and the decline threshold had to be reached before the individual’s last eGFR measurement, ensuring that this event occurred no later than death or study end (December 31, 2021). The timing of the eGFR decline was determined by estimating the date when the regression line crossed the 30% decline threshold. Dialysis and kidney transplantation were analyzed separately as secondary outcomes. In the time-to-event analysis, patients were followed from the index date until the first occurrence of a specific outcome, death, or study end, with virtually no loss to follow-up.

### Covariates

Baseline covariates included sociodemographics (age, sex, and calendar year of registration), eGFR, prior health care utilization (number of outpatient visits and hospitalizations during the past year), comorbidities, and concurrent use of medications. Definitions of comorbidities and concurrent medications are detailed in Table S1. All the covariates had complete information. We used the directed acyclic graphs to depict possible relationships among the exposures, measured covariates, unmeasured factors, and outcomes (Fig S2).

### Statistical Analyses

In the cross-sectional analysis, we reported the lifetime prevalence of SMI in patients with incident CKD, overall and stratified by sex or CKD stage. We calculated the standardized prevalence ratio^36^ to quantify the difference in the prevalence of SMI among the CKD population compared with the general population. The standardized prevalence ratio was the ratio of the observed number of SMI cases in the CKD population to the expected number based on age-, sex-, and calendar year–specific prevalence derived from the general population, with its 95% CI computed assuming a Poisson distribution. In the supplemental analyses, we reported the prevalence of schizophrenia spectrum disorders in patients with incident CKD as well as the prevalence of SMI in patients with either incident or prevalent CKD.

In the cohort analysis, we first examined the association between any SMI and health outcomes in patients with incident non-KRT CKD. Cox proportional hazard regression models, with age as the underlying time scale, were used to estimate hazard ratios (HRs) and 95% CIs for the associations. Specifically, the cause-specific hazards model was used to analyze 30% eGFR decline and initiation of KRT. We presented the minimally (age and sex) adjusted and multivariable-adjusted HRs. Subgroup analyses by sex were conducted, along with tests for multiplicative interaction. Furthermore, we investigated the relationship between each SMI and health outcomes, comparing CKD patients with a specific SMI to those without the corresponding SMI. In a sensitivity analysis, we used patients without any SMI as the reference group. Schizophrenia spectrum disorders were analyzed as a secondary exposure, and dialysis and kidney transplantation were examined separately as secondary outcomes. Statistical significance was defined as 2-tailed *P* < 0.05. All statistical analyses were performed using R software version 4.2.3 (R Foundation for Statistical Computing).

## Results

### Patient Characteristics

We identified 32,943 patients with incident CKD G3b-5 or undergoing KRT (Fig 1). The mean age was 70.4 ± 13.7 (SD) years, and 12,092 (36.7%) were women; the mean eGFR was 24.9 ± 8.5 mL/min/1.73 m^2^, and 8.6% were undergoing KRT (Table 1). Compared with CKD patients without SMI, those with any SMI were younger and more likely to be female. The distribution of stage of CKD and underlying cause of kidney disease varied by specific SMI. Compared with CKD patients without SMI, those with schizophrenia had a higher proportion of CKD G5 and KRT, and those with bipolar disorder had a higher proportion of CKD G3b. Lithium nephropathy was rarely observed in patients without SMI (0.1%), whereas it was recorded as the primary cause of kidney disease in 52.1% of patients with bipolar disorder, 12.0% of patients with schizophrenia, and 7.1% of patients with major depressive disorder.

### Prevalence of SMI

**Figure 2.**
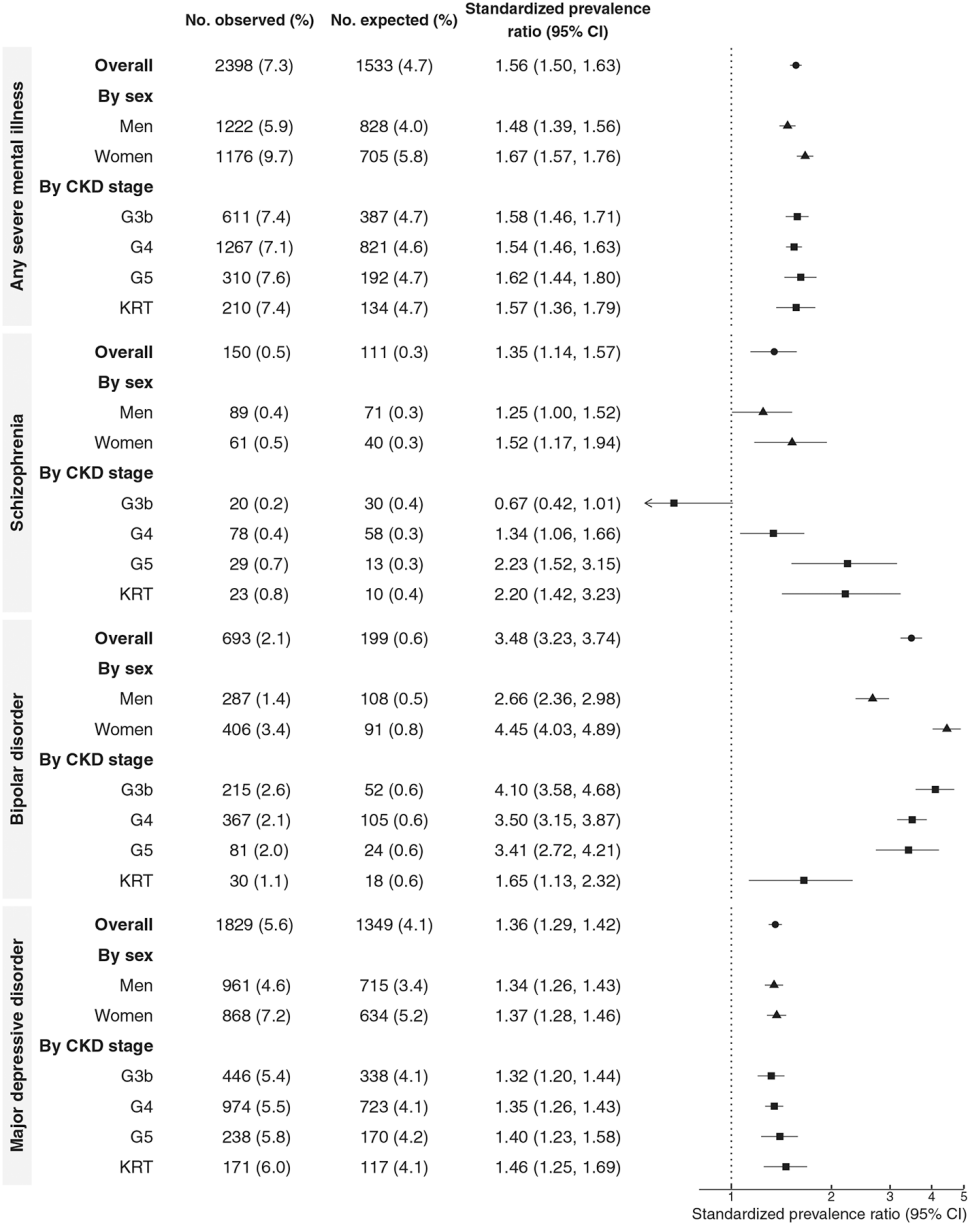
Lifetime prevalence of severe mental illness and standardized prevalence ratio in patients with incident CKD. Abbreviations: CI, confidence interval; KRT, kidney replacement therapy.

The prevalence of any SMI in patients with incident CKD was 7.3% (Fig 2). The observed number of SMI cases in patients with CKD was 56% higher than the expected number from the general population (standardized prevalence ratio, 1.56 [95% CI, 1.50–1.63]). The standardized prevalence ratio was slightly higher in women than men and largely similar across CKD stages.

For each specific SMI, the prevalence was 0.5% for schizophrenia, 2.1% for bipolar disorder, and 5.6% for major depressive disorder (Fig 2). Compared with the general population, the standardized prevalence ratio for schizophrenia, bipolar disorder, and major depressive disorder among patients with CKD was 1.35 (95% CI, 1.14–1.57), 3.48 (95% CI, 3.23–3.74), and 1.36 (95% CI, 1.29–1.42), respectively. The prevalence and standardized prevalence ratio of specific SMIs were generally higher in women than in men. Among patients on KRT, there was a higher standardized prevalence ratio for major depressive disorder and a lower estimate for bipolar disorder.

The prevalence of SMI was similar when patients with prevalent CKD were also included (Fig S3). The prevalence of schizophrenia spectrum disorders in patients with incident CKD was 1.4%, with a standardized prevalence ratio of 1.66 (95% CI, 1.51–1.82) (Fig S4).

### SMI and Health Outcomes

A total of 30,103 patients with incident non-KRT CKD were included in the following analyses. Baseline characteristics were presented in Table S2. Over a median follow-up period of 3.8 (IQR, 2.0–6.2) years, 8,465 individuals experienced a sustained 30% decline in eGFR, 7,304 individuals initiated KRT, and 14,864 deaths were recorded. Compared with CKD patients without SMI, those with any SMI had a higher rate of 30% decline in eGFR (HR, 1.13 [95% CI, 1.04–1.24]) and all-cause mortality (HR, 1.16 [95% CI, 1.08–1.24]), but a lower rate of initiating KRT (HR, 0.88 [95% CI, 0.80–0.97]) (Table 2). No apparent effect modification by sex was observed.

Each specific SMI, when compared with CKD patients without the corresponding SMI, was associated with a higher rate of all-cause mortality, with the HR being 2.12 (95% CI, 1.68–2.68) for schizophrenia, 1.13 (95% CI, 1.00–1.28) for bipolar disorder, and 1.09 (95% CI, 1.00–1.17) for major depressive disorder (Table 3). By contrast, each SMI was variably associated with kidney-related outcomes. Schizophrenia was not associated with a 30% decline in eGFR (HR, 0.92 [95% CI, 0.65–1.29]), and it was significantly associated with a lower rate of KRT initiation (HR, 0.56 [95% CI, 0.39–0.80]). Bipolar disorder was significantly associated with a higher rate of 30% decline in eGFR (HR, 1.47 [95% CI, 1.29–1.67]) but a lower rate of KRT initiation (HR, 0.79 [95% CI, 0.67–0.94]). Major depressive disorder was not associated with a 30% decline in eGFR (HR, 1.01 [95% CI, 0.91–1.12]) or initiation of KRT (HR, 0.95 [95% CI, 0.85–1.06]).

Similar associations were noted when comparing CKD patients with a specific SMI with those without any SMI (Table S3). Additionally, schizophrenia spectrum disorders were associated with a lower rate of KRT initiation (HR, 0.65 [95% CI, 0.53–0.81]) but a higher rate of 30% decline in eGFR (HR, 1.31 [95% CI, 1.10–1.57]) and mortality (HR, 1.69 [95% CI, 1.47–1.95]) (Table S4). Schizophrenia and bipolar disorder exhibited a stronger negative association with kidney transplantation than with dialysis (Table S5).

## Discussion

In this nationwide study, we report an overall prevalence of 7.3% for any SMI in patients with CKD. Specifically, the prevalence was 0.5% for schizophrenia, 2.1% for bipolar disorder, and 5.6% for major depressive disorder, representing an increase of 35%, 248%, and 36%, respectively, in comparison with the general population. Among CKD patients not undergoing KRT, all SMIs were associated with a higher mortality rate, and bipolar disorder was also associated with a faster eGFR decline. CKD patients with schizophrenia or bipolar disorder had a lower rate of initiating KRT.

Our finding of a higher prevalence of schizophrenia in patients with CKD echoes the earlier observation of a higher prevalence of kidney disease in patients with schizophrenia.^37^ Schizophrenia, compared with bipolar disorder and major depressive disorder, exhibited a stronger association with mortality, but we did not find an association with eGFR decline. Nonetheless, our baseline data indicated a higher percentage of stage G5 in CKD patients with schizophrenia.

Several risk factors for CKD have been identified in individuals with schizophrenia. First, patients with schizophrenia are at an increased risk for cardiometabolic conditions, including hypertension, diabetes, metabolic syndrome, and cardiovascular disease.^9,38^ Second, patients with schizophrenia are susceptible to medication non-adherence and unhealthy lifestyles such as smoking, poor diet, and physical inactivity.^38^ Third, many antipsychotics are associated with weight gain and metabolic side effects,^39^ although their direct impact on kidney health remains unclear.^40^ Finally, patients with schizophrenia face significant disparities in health care,^9,15^ and previous research has demonstrated inadequate predialysis nephrology care,^20,41^ such as fewer visits to nephrologists and suboptimal prescriptions. Physical illnesses, such as cardiovascular, metabolic, and infectious diseases have been highlighted as major contributors to the mortality gap observed in patients with SMI^9^ while kidney diseases have received relatively little attention. Our findings emphasize the interplay between SMI and CKD, shedding light on the importance of recognizing, understanding, and managing the coexistence of these conditions through a multidisciplinary care team.

Bipolar disorder is 3 times more prevalent in patients with CKD than in the general population, representing the largest difference among the 3 SMIs. Likewise, the relationship between bipolar disorder and CKD is influenced by a combination of biological, behavioral, and psychosocial determinants.^6^ In addition, lithium, which has been the mainstay of pharmacological treatment for bipolar disorder for decades, could be an important contributor to CKD. Although lithium nephrotoxicity has long been debated, recent evidence has suggested long-term use and supratherapeutic serum levels are associated with the development and progression of CKD.^42–45^ Our study showed that lithium nephropathy was recorded as the primary cause of kidney disease in half of patients with CKD and bipolar disorder, consistent with another Swedish study that validated the underlying cause using medical records.^46^

Besides a higher rate of mortality, we observed a faster eGFR decline for bipolar disorder. Most existing studies focused on lithium-induced nephropathy in patients with bipolar disorder; however, research has suggested that bipolar disorder per se might be a risk factor for the decline of kidney function, independent of lithium exposure.^42^ Prevention and management of the renal side effects of lithium treatment are complicated, but they remain a critical issue. Individuals with bipolar disorder who are at higher risk of subsequent eGFR decline may be identified before initiating lithium treatment using a prediction model based on age, sex, and baseline eGFR.^47^ Clinicians should carefully balance the relative risks and benefits when contemplating initiating or discontinuing lithium therapy, and ensure adequate monitoring and management of comorbidities in these patients.^48^

The prevalence of major depressive disorder in CKD patients from the current study is lower than that reported in a meta-analysis,^3^ which could be partially attributed to the absence of primary care data and patients’ under-reporting of depressive symptoms in routine care. However, register-based diagnosis may capture the patients in greater need of clinical care than approaches based on symptom measures. Our study found that a previous diagnosis of major depressive disorder in patients with CKD was associated with increased mortality, but not with eGFR decline or KRT initiation, whereas prior research mostly indicated significant associations between elevated depressive symptoms^17,18^ or incident diagnosis of major depressive disorder^19^ and adverse kidney outcomes. A plausible explanation for the contrasting finding is the episodic nature of major depressive disorder, implying that individuals may have achieved remission or recovery by their time of cohort entry. Moreover, given the potential bidirectional relationship between major depressive disorder and CKD,^49^ depressive episodes occurring during the follow-up period may also attenuate the association. Of note, these characteristics differ from schizophrenia and bipolar disorder, which typically manifest in early adulthood, preceding the development of CKD, and are more perceived as lifelong conditions requiring ongoing care and support.^50,51^

Despite a poorer prognosis in CKD patients with schizophrenia/bipolar disorder, they exhibited a significantly lower rate of initiating KRT. A previous cross-sectional study similarly observed a lower proportion of CKD patients with schizophrenia to receive KRT,^52^ whereas 2 studies conducted in CKD-free populations showed opposite associations between SMI and the receipt of KRT.^20,41^ The decision to initiate KRT takes into account important nonkidney factors, such as the patient’s preferences, pre-existing conditions, and overall goals of care.^53^ In particular, maintenance dialysis requires a high degree of patient cooperation to achieve favorable outcomes, involving adherence to dialysis sessions, prescribed medications, and fluid/dietary restrictions.^54^ Affective and psychotic disorders have historically been considered as relative or absolute contraindications to transplantation because of concerns about medical adherence and poor postoperative outcomes.^55^

Our findings do not necessarily indicate unequal access to KRT, but they still highlight its possible underutilization in patients with SMI, pointing to the need to understand the underlying decision-making processes. There have been considerable efforts in the nephrology community to enhance patient-centered care and outcomes over the past decade,^56^ but mental health remains underrecognized. Identifying the presence of SMI provides the dialysis/transplant center an opportunity to recommend or offer appropriate treatment and additional support, in collaboration with other health care teams, to address the potential barriers and optimize health outcomes.^57^

The strengths of our study include a large nationwide cohort of referred well-phenotyped CKD patients, analysis of major SMIs across CKD stages, and the setting involving real-world patients from a country with universal tax-funded health care, which mitigates selection bias from disparate access to health care.

Several limitations should be acknowledged. First, misclassification bias exists because the identification of SMI relied on diagnoses from inpatient or specialist outpatient care. The lack of primary care diagnoses may underestimate the prevalence of SMI, particularly for major depressive disorder. However, patients with SMI who exclusively utilize primary care services without engagement in outpatient or inpatient care during an observation period exceeding 10 years are likely few. Second, ascertainment bias may arise because patients with SMIs or CKD encounter physicians more frequently, leading to the identification of other conditions and an overrepresentation of SMI in CKD patients. The closer monitoring could also result in a slight overestimation of association with eGFR decline. Third, given the observational nature, our study cannot establish causal relationships between SMI and clinical outcomes because residual and unmeasured confounding can never be ruled out. Fourth, the probability of false-positive discovery increased due to multiple comparisons, so findings from our analyses should be interpreted as exploratory. Fifth, the Swedish Renal Registry did not routinely enroll patients with CKD G1–3a; future studies are needed to determine whether our findings can be extended to earlier stages of CKD. Finally, our data reflect Swedish clinical practice, and generalization to other settings should be made with caution.

To conclude, this study highlights a higher prevalence of SMI in patients with CKD relative to the general population. SMI is associated with a higher mortality rate in patients with CKD, and bipolar disorder is associated with a faster eGFR decline. However, CKD patients with schizophrenia or bipolar disorder are less likely to initiate KRT. Recognizing the presence of SMI in patients with CKD needs to be emphasized, which should not be a barrier to accessing kidney care but rather an opportunity to improve health outcomes through the provision of appropriate treatment and support via multidisciplinary team care.

## Supplementary Material

1

**Figure S1:** Graphical depiction of data coverage and measurement.

**Figure S2:** Potential directed acyclic diagrams for the association between severe mental illness and health outcomes.

**Figure S3:** Lifetime prevalence of severe mental illness and standardized prevalence ratio in patients with incident or prevalent CKD (N = 48,907).

**Figure S4:** Lifetime prevalence of schizophrenia spectrum disorders and standardized prevalence ratio in patients with incident CKD (N = 32,943).

**Table S1:** Definition of comorbidities and concurrent medications.

**Table S2:** Baseline characteristics of patients with incident non-KRT CKD.

**Table S3:** Association between each severe mental illness versus no severe mental illness and health outcomes in patients with incident non-KRT CKD (N = 30,103).

**Table S4:** Association between schizophrenia spectrum disorders and health outcomes in patients with incident non-KRT CKD (N = 30,103).

**Table S5:** Association between each severe mental illness and dialysis/kidney transplantation in patients with incident non-KRT CKD (N = 30,103).

## Figures and Tables

**Table 1.** Baseline Characteristics of Patients With Incident CKD

Characteristics	Overall N = 32,943	No Severe Mental Illness N = 30,545	Any Severe Mental Illness N = 2,398	Schizophrenia N = 150	Bipolar Disorder N = 693	Major Depressive Disorder N = 1,829
Age, y	70.4 ± 13.7	70.7 ± 13.7	66.2 ± 13.5	61.5 ± 11.2	67.2 ± 10.3	66.1 ± 14.4
Female	12,092 (36.7%)	10,916 (35.7%)	1,176 (49.0%)	61 (40.7%)	406 (58.6%)	868 (47.5%)
Calendar year						
2010–2012	9,033 (27.4%)	8,458 (27.7%)	575 (24.0%)	44 (29.3%)	179 (25.8%)	406 (22.2%)
2013–2016	12,051 (36.6%)	11,164 (36.5%)	887 (37.0%)	49 (32.7%)	264 (38.1%)	677 (37.0%)
2017–2020	11,859 (36.0%)	10,923 (35.8%)	936 (39.0%)	57 (38.0%)	250 (36.1%)	746 (40.8%)
eGFR, mL/min/1.73 m^2,a^	24.9 ± 8.5	24.9 ± 8.5	25.0 ± 8.8	22.1 ± 8.5	25.7 ± 8.6	24.9 ± 8.7
Stage of CKD						
G3b	8,246 (25.0%)	7,635 (25.0%)	611 (25.5%)	20 (13.3%)	215 (31.0%)	446 (24.4%)
G4	17,779 (54.0%)	16,512 (54.1%)	1,267 (52.8%)	78 (52.0%)	367 (53.0%)	974 (53.3%)
G5	4,083 (12.4%)	3,773 (12.4%)	310 (12.9%)	29 (19.3%)	81 (11.7%)	238 (13.0%)
KRT	2,835 (8.6%)	2,625 (8.6%)	210 (8.8%)	23 (15.3%)	30 (4.3%)	171 (9.3%)
Cause of kidney disease						
Hypertensive/renovascular nephropathy	9,637 (29.3%)	9,150 (30.0%)	487 (20.3%)	21 (14.0%)	74 (10.7%)	428 (23.4%)
Diabetic kidney disease	6,805 (20.7%)	6,313 (20.7%)	492 (20.5%)	28 (18.7%)	60 (8.7%)	432 (23.6%)
Glomerulonephritis	2,573 (7.8%)	2,422 (7.9%)	151 (6.3%)	17 (11.3%)	16 (2.3%)	129 (7.1%)
Polycystic kidney disease	1,212 (3.7%)	1,148 (3.8%)	64 (2.7%)	4 (2.7%)	23 (3.3%)	46 (2.5%)
Lithium nephropathy	416 (1.3%)	29 (0.1%)	387 (16.1%)	18 (12.0%)	361 (52.1%)	130 (7.1%)
Other specified causes	8,851 (26.9%)	8,260 (27.0%)	591 (24.6%)	43 (28.7%)	109 (15.7%)	491 (26.8%)
Undefined nephropathy	3,449 (10.5%)	3,223 (10.6%)	226 (9.4%)	19 (12.7%)	50 (7.2%)	173 (9.5%)

Values are given as mean ± SD for continuous variables and as number (percentage) for categorical variables. Abbreviations: CKD, chronic kidney disease; eGFR, estimated glomerular filtration rate; KRT, kidney replacement therapy.

a Mean eGFR was reported among patients with non-KRT CKD.

**Table 2.** Association Between Any Severe Mental Illness and Health Outcomes in Patients With Incident Non-KRT CKD (N = 30,103)

	Any Severe Mental Illness		No Severe Mental Illness		Hazard Ratio (95% CI)		
	No. of Events	Incidence Rate per 1,000 PY	No. of Events	Incidence rate per 1,000 PY	Age and Sex Adjusted	Multivariable Adjusted^a^	*P*_interaction_
**30**% **Decline in eGFR**							
Overall	616	86.5	7,849	83.9	0.97 (0.89–1.05)	1.13 (1.04–1.24)	
Men	292	85.1	5,240	90.2	0.86 (0.77–0.97)	1.04 (0.92–1.18)	0.003
Women	324	87.8	2,609	73.5	1.09 (0.97–1.23)	1.22 (1.08–1.38)	
**Initiation of KRT**							
Overall	521	68.0	6,783	68.2	0.86 (0.78–0.94)	0.88 (0.80–0.97)	
Men	290	81.7	4,559	73.6	0.92 (0.82–1.03)	0.96 (0.85–1.09)	0.3
Women	231	56.2	2,224	59.3	0.78 (0.68–0.90)	0.78 (0.68–0.90)	
**All-Cause Mortality**							
Overall	1,028	111.2	13,836	113.7	1.34 (1.25–1.42)	1.16 (1.08–1.24)	
Men	545	122.4	9,094	118.5	1.43 (1.31–1.56)	1.22 (1.11–1.34)	0.2
Women	483	100.8	4,742	105.4	1.23 (1.12–1.35)	1.09 (0.99–1.21)	

Abbreviations: CKD, chronic kidney disease; eGFR, estimated glomerular filtration rate; KRT, kidney replacement therapy; PY, person-years.

a Models were adjusted for age, sex, calendar year, baseline eGFR, prior health care use, physical comorbidities (hypertension, diabetes mellitus, myocardial infarction, atrial fibrillation, stroke, congestive heart failure, peripheral vascular disease, cancer, lung disease, and liver disease), neuropsychiatric comorbidities (dementia, substance use disorders, and anxiety disorders), and concurrent medications (renin-angiotensin system inhibitors, β-blockers, calcium channel blockers, diuretics, statins, antiplatelet drugs, and nonsteroidal anti-inflammatory drugs).

**Table 3.** Association Between Each Severe Mental Illness and Health Outcomes in Patients With Incident Non-KRT CKD (N = 30,103)

	Severe Mental Illness		No Corresponding Severe Mental Illness^a^		Hazard Ratio (95% CI)	
	No. of Events	Incidence Rate per 1,000 PY	No. of Events	Incidence Rate per 1,000 PY	Age and Sex Adjusted	Multivariable Adjusted^b^
**Schizophrenia**						
30% decline in eGFR	33	85.4	8,432	84.1	0.87 (0.62–1.23)	0.92 (0.65–1.29)
Initiation of KRT	30	74.3	7,274	68.2	0.80 (0.56–1.15)	0.56 (0.39–0.80)
All-cause mortality	72	146.3	14,792	113.4	2.47 (1.95–3.11)	2.12 (1.68–2.68)
**Bipolar Disorder**						
30% decline in eGFR	244	106.7	8,221	83.5	1.25 (1.10–1.43)	1.47 (1.29–1.67)
Initiation of KRT	140	50.7	7,164	68.6	0.69 (0.58–0.82)	0.79 (0.67–0.94)
All-cause mortality	270	84.7	14,594	114.2	1.01 (0.89–1.13)	1.13 (1.00–1.28)
**Major Depressive Disorder**						
30% decline in eGFR	416	78.1	8,049	84.4	0.86 (0.78–0.95)	1.01 (0.91–1.12)
Initiation of KRT	409	74.7	6,895	67.8	0.94 (0.85–1.04)	0.95 (0.85–1.06)
All-cause mortality	789	117.6	14,075	113.3	1.38 (1.28–1.48)	1.09 (1.00–1.17)

Abbreviations: CKD, chronic kidney disease; eGFR, estimated glomerular filtration rate; KRT, kidney replacement therapy; PY, person-years.

a For each exposure, the reference group consisted of CKD patients without the corresponding severe mental illness.

b Models were adjusted for age, sex, calendar year, baseline eGFR, prior health care use, physical comorbidities (hypertension, diabetes mellitus, myocardial infarction, atrial fibrillation, stroke, congestive heart failure, peripheral vascular disease, cancer, lung disease, and liver disease), neuropsychiatric comorbidities (dementia, substance use disorders, and anxiety disorders), and concurrent medications (renin-angiotensin system inhibitors, β-blockers, calcium channel blockers, diuretics, statins, antiplatelet drugs, and nonsteroidal anti-inflammatory drugs).

## Data Availability

Data will be available on reasonable request by submitting a protocol to the Swedish Renal Registry. For inquiries about data access, please contact Dr Evans at marie.evans@ki.se.
