# Supplementary material for: Prevalence of Severe Mental Illness and Its Associations With Health Outcomes in Patients With CKD: A Swedish Nationwide Study
Source: Am J Kidney Dis. Author manuscript; Available in PMC 2026 May 12. (PMC13166082; doi:10.1053/j.ajkd.2024.12.004)
Supplement: 1 [file NIHMS2171777-supplement-1.pdf]

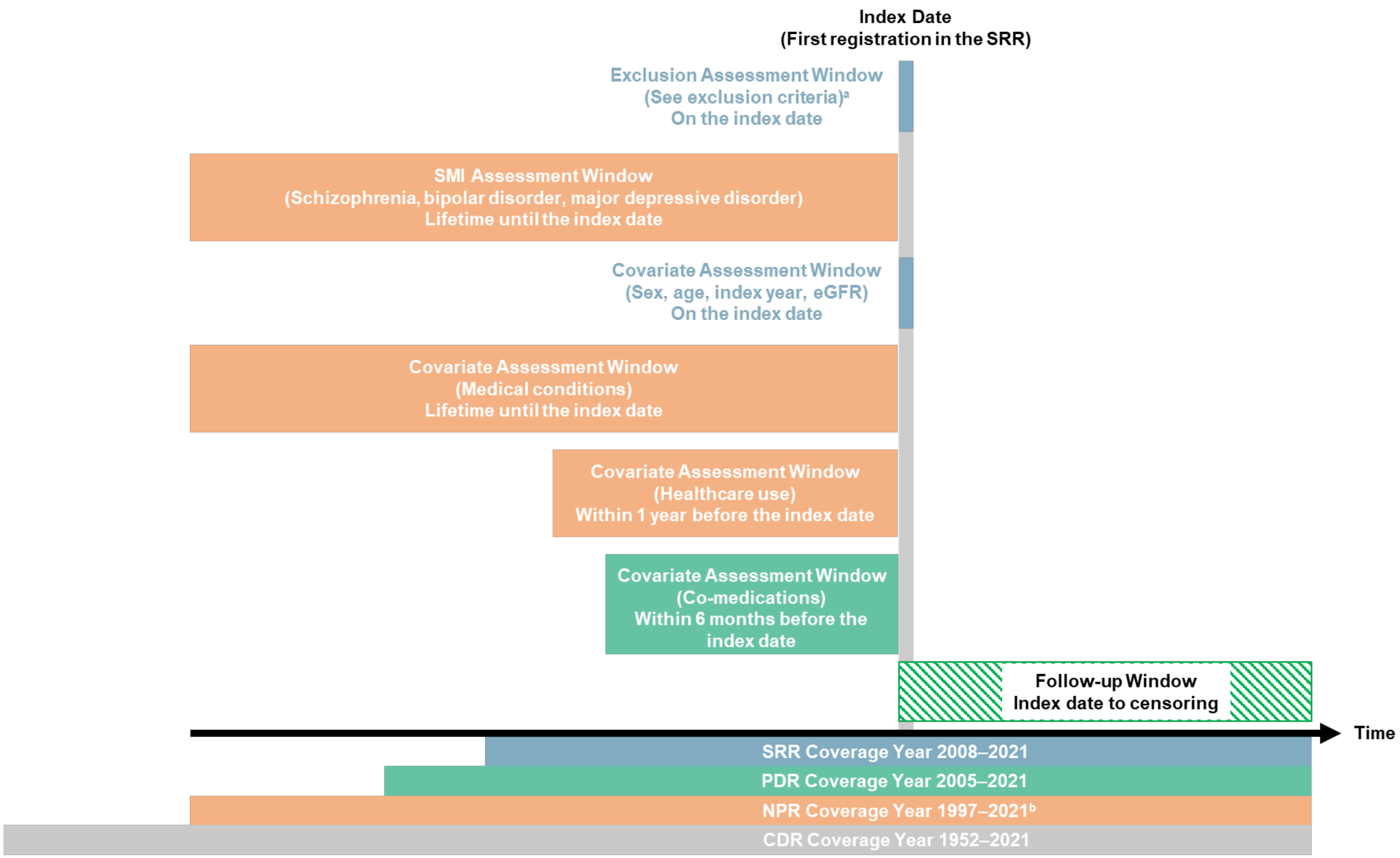

Figure S1. Graphical depiction of data coverage and measurement

Abbreviations: CDR, Cause of Death Register; eGFR, estimated glomerular filtration rate; NPR, National Patient Register; PDR, Prescribed Drug Register; SMI, severe mental illness; SRR, Swedish Renal Registry.

[a] Exclusion criteria included age <18 years, missing eGFR, eGFR >45 mL/min/1.73 m^2, and first registration before 2010.

[b] The National Patient Register provides ICD-10 coded clinical diagnoses for inpatient care since 1997 and outpatient specialist care since 2001.

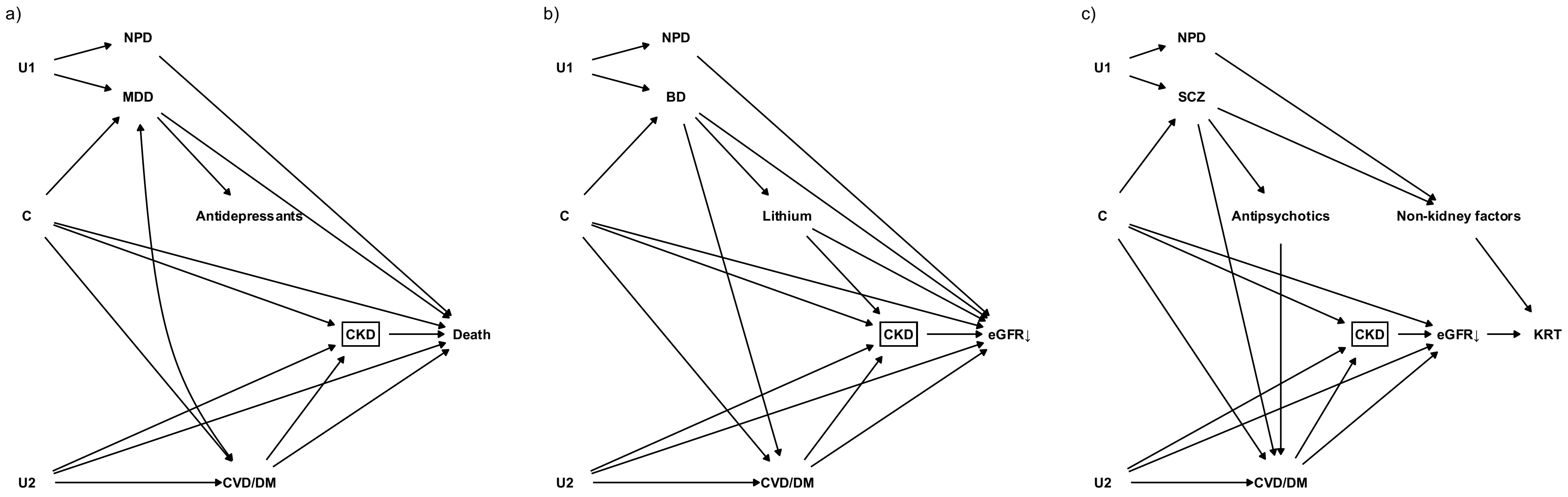

Figure S2. Potential directed acyclic diagrams for the association between severe mental illness and health outcomes

Abbreviations: BD, bipolar disorder; CVD, cardiovascular disease; DM, diabetes mellitus; eGFR, estimated glomerular filtration rate; KRT, kidney replacement therapy; MDD, major depressive disorder; NPD, other neuropsychiatric disorder; SCZ, schizophrenia.

Figure S2-a depicts the relationship between major depressive disorder and death; Figure S2-b depicts the relationship between bipolar disorder and 30% decline in eGFR; Figure S2-c depicts the relationship between schizophrenia and initiation of KRT. "C" represents common causes of the specific outcome and several medical conditions, such as socio-demographics (e.g., age and sex) and general health status (e.g., prior healthcare utilization, comorbidities, and concurrent medications). "U1" represents unmeasured common causes of mental disorders. "U2" represents unmeasured common causes of somatic disorders. "Rectangular around CKD" represents that the study population is restricted to patients with CKD.

		No. observed (%)	No. expected (%)	Standardized prevalence ratio (95% CI)
Any severe mental illness	**Overall**	3403 (7.0)	2122 (4.3)	1.60 (1.55, 1.66)
	**By sex**			
	Men	1750 (5.6)	1156 (3.7)	1.51 (1.44, 1.59)
	Women	1653 (9.3)	966 (5.4)	1.71 (1.63, 1.79)
	**By CKD stage**			
	G3b	837 (7.3)	523 (4.6)	1.60 (1.49, 1.71)
	G4	1569 (6.9)	1003 (4.4)	1.56 (1.49, 1.64)
	G5	452 (7.0)	279 (4.3)	1.62 (1.47, 1.77)
	KRT	545 (6.6)	317 (3.8)	1.72 (1.58, 1.87)
Schizophrenia	**Overall**	223 (0.5)	166 (0.3)	1.34 (1.17, 1.53)
	**By sex**			
	Men	130 (0.4)	108 (0.3)	1.20 (1.01, 1.42)
	Women	93 (0.5)	58 (0.3)	1.60 (1.29, 1.94)
	**By CKD stage**			
	G3b	35 (0.3)	42 (0.4)	0.83 (0.59, 1.14)
	G4	97 (0.4)	75 (0.3)	1.30 (1.06, 1.57)
	G5	43 (0.7)	21 (0.3)	2.10 (1.53, 2.79)
	KRT	48 (0.6)	29 (0.3)	1.66 (1.23, 2.17)
Bipolar disorder	**Overall**	913 (1.9)	278 (0.6)	3.28 (3.07, 3.50)
	**By sex**			
	Men	375 (1.2)	152 (0.5)	2.47 (2.23, 2.73)
	Women	538 (3.0)	126 (0.7)	4.26 (3.91, 4.63)
	**By CKD stage**			
	G3b	256 (2.2)	71 (0.6)	3.58 (3.16, 4.04)
	G4	442 (1.9)	129 (0.6)	3.43 (3.12, 3.76)
	G5	125 (1.9)	35 (0.5)	3.57 (2.98, 4.23)
	KRT	90 (1.1)	43 (0.5)	2.11 (1.70, 2.57)
Major depressive disorder	**Overall**	2633 (5.4)	1848 (3.8)	1.42 (1.37, 1.48)
	**By sex**			
	Men	1399 (4.5)	986 (3.2)	1.42 (1.35, 1.49)
	Women	1234 (6.9)	862 (4.9)	1.43 (1.35, 1.51)
	**By CKD stage**			
	G3b	638 (5.6)	455 (4.0)	1.40 (1.30, 1.51)
	G4	1205 (5.3)	879 (3.9)	1.37 (1.30, 1.45)
	G5	340 (5.3)	245 (3.8)	1.39 (1.25, 1.54)
	KRT	450 (5.4)	270 (3.3)	1.67 (1.52, 1.83)

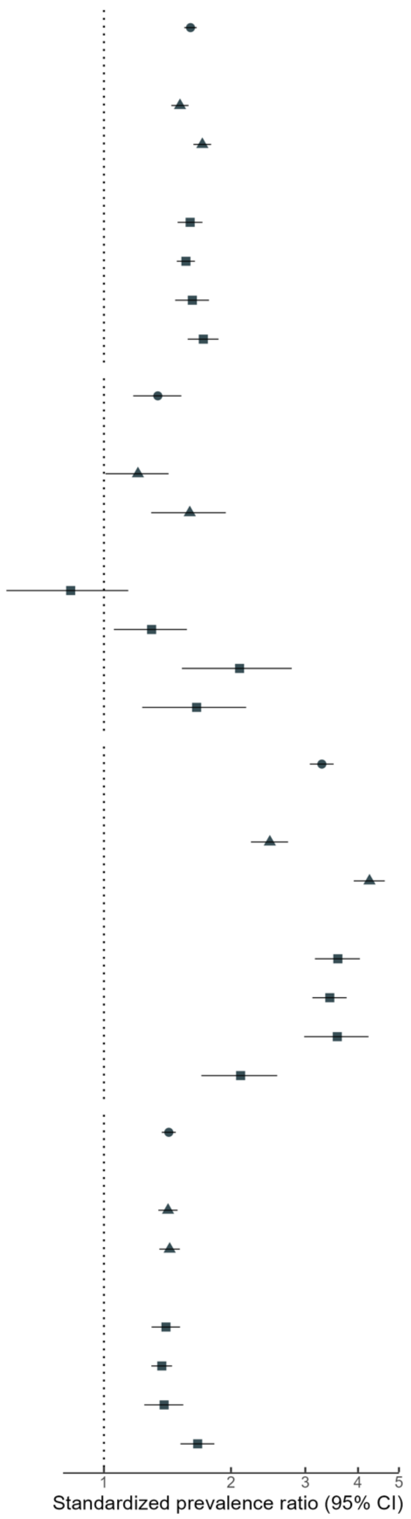

Figure S3. Lifetime prevalence of severe mental illness and standardized prevalence ratio in patients with incident or prevalent CKD (N = 48,907)

Abbreviations: CI, confidence interval; CKD, chronic kidney disease; KRT, kidney replacement therapy.

		No. observed (%)	No. expected (%)	Standardized prevalence ratio (95% CI)
Any severe mental illness*	**Overall**	2524 (7.7)	1618 (4.9)	1.56 (1.50, 1.62)
	**By sex**			
	Men	1284 (6.2)	873 (4.2)	1.47 (1.39, 1.55)
	Women	1240 (10.3)	745 (6.2)	1.66 (1.57, 1.76)
	**By CKD stage**			
	G3b	640 (7.8)	408 (4.9)	1.57 (1.45, 1.69)
	G4	1324 (7.4)	867 (4.9)	1.53 (1.45, 1.61)
	G5	335 (8.2)	203 (5.0)	1.65 (1.48, 1.84)
	KRT	225 (7.9)	141 (5.0)	1.59 (1.39, 1.81)
Schizophrenia spectrum disorders	**Overall**	445 (1.4)	267 (0.8)	1.66 (1.51, 1.82)
	**By sex**			
	Men	211 (1.0)	151 (0.7)	1.40 (1.22, 1.59)
	Women	234 (1.9)	116 (1.0)	2.01 (1.77, 2.28)
	**By CKD stage**			
	G3b	88 (1.1)	69 (0.8)	1.28 (1.03, 1.57)
	G4	226 (1.3)	142 (0.8)	1.59 (1.40, 1.81)
	G5	81 (2.0)	33 (0.8)	2.49 (1.99, 3.07)
	KRT	50 (1.8)	24 (0.9)	2.06 (1.54, 2.68)

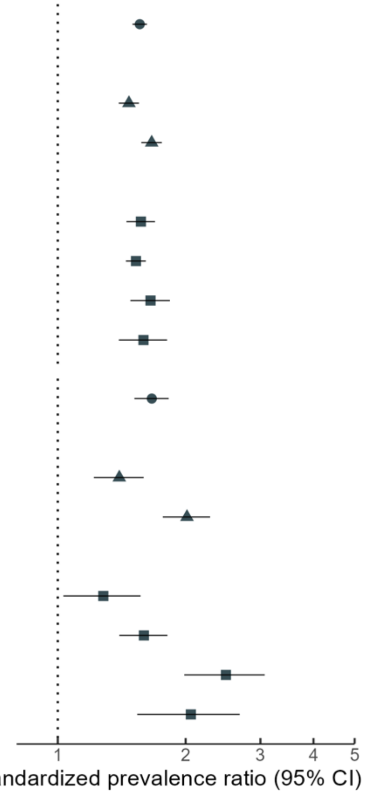

Figure S4. Lifetime prevalence of schizophrenia spectrum disorders and standardized prevalence ratio in patients with incident CKD (N = 32,943)

Abbreviations: CI, confidence interval; CKD, chronic kidney disease; KRT, kidney replacement therapy.

*Any severe mental illness included schizophrenia spectrum disorders, bipolar disorder, and major depressive disorder.

Table S1. Definition of comorbidities and concurrent medications

	ICD-10 codes	ATC codes
**Comorbidity (anytime before baseline)**		
Comorbid physical illnesses		
Hypertension	I10–I15	C03, C07, C08, C09
Diabetes mellitus	E10–E14	A10
Cardiovascular disease		
Myocardial infarction	I21, I22, I25.2	
Stroke	G45, I60–I64, I67, I69	
Atrial fibrillation	I48	
Congestive heart failure	I11.0, I13.0, I13.2, I25.5, I42.0, I42.6–I42.9, I43, I50	
Peripheral vascular disease	I70, I71, I73.1, I73.8, I73.9, I77.1, I79.0, I79.2, K55	
Cancer (excluding nonmelanoma skin cancer)	C00–C97, excluding C44	
Lung disease	J41–J47, J60–J70	
Liver disease	B15–B19, K70– K77, I85.0, I85.9, I98.2, I98.3	
Comorbid neuropsychiatric illnesses		
Dementia	F00–F03, F05.1, G30, G31.1, G31.9	
Substance use disorders	F10–F19	
Anxiety disorders	F40, F41	
**Concurrent medications (within 6 months before baseline)**		
Renin-angiotensin system inhibitors		C09
Beta-blockers		C07
Calcium channel blockers		C08
Diuretics		C03
Statins		C10AA
Antiplatelet drugs		B01AC
Non-steroidal anti-inflammatory drugs		M01A

Abbreviations: ATC, anatomical therapeutic chemical; ICD-10, international classification of diseases, tenth revision.

Table S2. Baseline characteristics of patients with incident non-KRT CKD

Characteristics	No severe mental illness N = 27,916	Any severe mental illness N = 2187	Schizophrenia N = 127	Bipolar disorder N = 663	Major depressive disorder N = 1657
**Age, years, mean ± SD**	71.4 ± 13.1	66.8 ± 13.2	62.7 ± 10.8	67.3 ± 10.0	66.7 ± 14.1
**Female, n (%)**	10,019 (35.9)	1096 (50.1)	58 (45.7)	394 (59.4)	801 (48.3)
**Calendar year, n (%)**					
2010–2012	7541 (27.0)	505 (23.1)	35 (27.6)	169 (25.5)	353 (21.3)
2013–2016	10,246 (36.7)	809 (37.0)	39 (30.7)	257 (38.8)	611 (36.9)
2017–2020	10,129 (36.3)	873 (39.9)	53 (41.7)	237 (35.7)	693 (41.8)
**eGFR, mL/min/1.73 m^2, mean ± SD**	24.9 ± 8.5	25.0 ± 8.8	22.1 ± 8.5	25.7 ± 8.6	24.9 ± 8.7
**Stage of CKD, n (%)**					
G3b	7633 (27.3)	610 (27.9)	20 (15.7)	215 (32.4)	445 (26.9)
G4	16,512 (59.1)	1267 (57.9)	78 (61.4)	367 (55.4)	974 (58.8)
G5	3771 (13.5)	310 (14.2)	29 (22.8)	81 (12.2)	238 (14.4)
**Cause of kidney disease, n (%)**					
Hypertensive/renovascular nephropathy	8764 (31.4)	458 (20.9)	18 (14.2)	72 (10.9)	404 (24.4)
Diabetic kidney disease	5908 (21.2)	448 (20.5)	28 (22.0)	58 (8.7)	389 (23.5)
Glomerulonephritis	2114 (7.6)	127 (5.8)	14 (11.0)	11 (1.7)	111 (6.7)
Lithium nephropathy	28 (0.1)	377 (17.2)	17 (13.4)	351 (52.9)	128 (7.7)
Other specified causes	8267 (29.6)	578 (26.4)	35 (27.6)	122 (18.4)	474 (28.6)
Undefined nephropathy	2835 (10.2)	199 (9.1)	15 (11.8)	49 (7.4)	151 (9.1)
**Healthcare utilization during the past year, mean ± SD**					
Number of hospitalizations	1.2 ± 1.8	1.7 ± 2.4	2.0 ± 2.5	1.2 ± 2.0	1.9 ± 2.5
Number of outpatient visits	4.9 ± 4.3	6.5 ± 5.4	5.5 ± 4.1	5.9 ± 4.8	6.9 ± 5.7

Characteristics	No severe mental illness N = 27,916	Any severe mental illness N = 2187	Schizophrenia N = 127	Bipolar disorder N = 663	Major depressive disorder N = 1657
**Comorbidities, n (%)**					
Hypertension	26,774 (95.9)	1997 (91.3)	112 (88.2)	558 (84.2)	1545 (93.2)
Diabetes mellitus	12,014 (43.0)	944 (43.2)	61 (48.0)	207 (31.2)	772 (46.6)
Myocardial infarction	5371 (19.2)	329 (15.0)	19 (15.0)	48 (7.2)	291 (17.6)
Atrial fibrillation	6180 (22.1)	324 (14.8)	10 (7.9)	51 (7.7)	288 (17.4)
Stroke	4444 (15.9)	412 (18.8)	19 (15.0)	75 (11.3)	356 (21.5)
Congestive heart failure	7642 (27.4)	508 (23.2)	28 (22.0)	77 (11.6)	440 (26.6)
Peripheral vascular disease	4008 (14.4)	264 (12.1)	15 (11.8)	29 (4.4)	235 (14.2)
Cancer	5826 (20.9)	397 (18.2)	10 (7.9)	96 (14.5)	333 (20.1)
Lung disease	3793 (13.6)	367 (16.8)	18 (14.2)	79 (11.9)	312 (18.8)
Liver disease	1052 (3.8)	157 (7.2)	<10	17 (2.6)	145 (8.8)
Dementia	402 (1.4)	107 (4.9)	<10	32 (4.8)	90 (5.4)
Substance use disorders	1494 (5.4)	426 (19.5)	21 (16.5)	91 (13.7)	370 (22.3)
Anxiety disorders	539 (1.9)	504 (23.0)	18 (14.2)	104 (15.7)	455 (27.5)
**Concurrent medications, n (%)**					
Renin-angiotensin system inhibitors	19,022 (68.1)	1169 (53.5)	70 (55.1)	263 (39.7)	954 (57.6)
Beta-blockers	18,012 (64.5)	1225 (56.0)	70 (55.1)	291 (43.9)	982 (59.3)
Calcium channel blockers	16,283 (58.3)	1117 (51.1)	74 (58.3)	300 (45.2)	860 (51.9)
Diuretics	17,885 (64.1)	1236 (56.5)	72 (56.7)	265 (40.0)	1013 (61.1)
Statins	14,914 (53.4)	1021 (46.7)	60 (47.2)	250 (37.7)	815 (49.2)
Antiplatelet drugs	10,855 (38.9)	742 (33.9)	43 (33.9)	155 (23.4)	616 (37.2)
Non-steroidal anti-inflammatory drugs	1375 (4.9)	130 (5.9)	<10	33 (5.0)	104 (6.3)

Abbreviations: CKD, chronic kidney disease; eGFR, estimated glomerular filtration rate; SD, standard deviation.

Table S3. Association between each severe mental illness versus no severe mental illness and health outcomes in patients with incident non-KRT CKD (N = 30,103)

	Severe mental illness		No severe mental illness[a]		Hazard ratio (95% CI)	
	No. of events	Event rate per 1000 person-years	No. of events	Event rate per 1000 person-years	Age- and sex-adjusted	Multivariable adjusted
**Schizophrenia**						
30% decline in eGFR	33	85.4	7849	83.9	0.87 (0.62, 1.22)	0.94 (0.67, 1.32)
Initiation of KRT	30	74.3	6783	68.2	0.80 (0.56, 1.14)	0.55 (0.39, 0.79)
All-cause mortality	72	146.3	13,836	113.7	2.53 (2.00, 3.19)	2.14 (1.69, 2.70)
**Bipolar disorder**						
30% decline in eGFR	244	106.7	7849	83.9	1.24 (1.09, 1.41)	1.48 (1.30, 1.69)
Initiation of KRT	140	50.7	6783	68.2	0.69 (0.58, 0.81)	0.78 (0.66, 0.93)
All-cause mortality	270	84.7	13,836	113.7	1.03 (0.91, 1.16)	1.13 (1.00, 1.28)
**Major depressive disorder**						
30% decline in eGFR	416	78.1	7849	83.9	0.87 (0.79, 0.96)	1.01 (0.91, 1.13)
Initiation of KRT	409	74.7	6783	68.2	0.93 (0.84, 1.03)	0.94 (0.84, 1.05)
All-cause mortality	789	117.6	13,836	113.7	1.39 (1.29, 1.49)	1.10 (1.02, 1.19)

Abbreviations: CI, confidence interval; CKD, chronic kidney disease; eGFR, estimated glomerular filtration rate; KRT, kidney replacement therapy.

[a] A common reference group consisted of patients without any of the three severe mental illnesses.

[b] Models were adjusted for age, sex, calendar year, baseline eGFR, prior healthcare use, physical comorbidities (hypertension, diabetes mellitus, myocardial infarction, atrial fibrillation, stroke, congestive heart failure, peripheral vascular disease, cancer, lung disease, and liver disease), neuropsychiatric comorbidities (dementia, substance use disorders, and anxiety disorders), and concurrent medications (renin-angiotensin system inhibitors, beta-blockers, calcium channel blockers, diuretics, statins, antiplatelet drugs, and non-steroidal anti-inflammatory drugs).

Table S4. Association between schizophrenia spectrum disorders and health outcomes in patients with incident non-KRT CKD (N = 30,103)

	Schizophrenia spectrum disorders		No schizophrenia spectrum disorders		Hazard ratio (95% CI)	
	No. of events	Event rate per 1000 person-years	No. of events	Event rate per 1000 person-years	Age- and sex-adjusted	Multivariable adjusted*
30% decline in eGFR	129	109.1	8336	83.8	1.17 (0.98, 1.39)	1.31 (1.10, 1.57)
Initiation of KRT	91	67.3	7213	68.2	0.76 (0.62, 0.94)	0.65 (0.53, 0.81)
All-cause mortality	199	122.8	14,665	113.4	1.80 (1.57, 2.08)	1.69 (1.47, 1.95)

Abbreviations: CI, confidence interval; CKD, chronic kidney disease; eGFR, estimated glomerular filtration rate; KRT, kidney replacement therapy.

*Models were adjusted for age, sex, calendar year, baseline eGFR, prior healthcare use, physical comorbidities (hypertension, diabetes mellitus, myocardial infarction, atrial fibrillation, stroke, congestive heart failure, peripheral vascular disease, cancer, lung disease, and liver disease), neuropsychiatric comorbidities (dementia, substance use disorders, and anxiety disorders), and concurrent medications (renin-angiotensin system inhibitors, beta-blockers, calcium channel blockers, diuretics, statins, antiplatelet drugs, and non-steroidal anti-inflammatory drugs).

Table S5. Association between each severe mental illness and dialysis/kidney transplantation in patients with incident non-KRT CKD (N = 30,103)

	Severe mental illness		No severe mental illness		Hazard ratio (95% CI)	
	No. of events	Event rate per 1000 person-years	No. of events	Event rate per 1000 person-years	Age- and sex-adjusted	Multivariable adjusted*
**Schizophrenia**						
Dialysis	29	71.6	6765	62.1	0.89 (0.62, 1.29)	0.63 (0.44, 0.91)
Kidney transplantation	<10	8.3	1908	15.5	0.29 (0.11, 0.78)	0.27 (0.10, 0.72)
**Bipolar disorder**						
Dialysis	129	46.2	6665	62.5	0.69 (0.58, 0.83)	0.81 (0.68, 0.97)
Kidney transplantation	31	10.0	1881	15.7	0.60 (0.42, 0.86)	0.64 (0.45, 0.92)
**Major depressive disorder**						
Dialysis	389	70.2	6405	61.7	1.01 (0.91, 1.12)	0.97 (0.87, 1.09)
Kidney transplantation	105	16.5	1807	15.5	0.68 (0.56, 0.83)	1.08 (0.87, 1.33)

Abbreviations: CI, confidence interval; CKD, chronic kidney disease; KRT, kidney replacement therapy.

*Models were adjusted for age, sex, calendar year, baseline eGFR, prior healthcare use, physical comorbidities (hypertension, diabetes mellitus, myocardial infarction, atrial fibrillation, stroke, congestive heart failure, peripheral vascular disease, cancer, lung disease, and liver disease), neuropsychiatric comorbidities (dementia, substance use disorders, and anxiety disorders), and concurrent medications (renin-angiotensin system inhibitors, beta-blockers, calcium channel blockers, diuretics, statins, antiplatelet drugs, and non-steroidal anti-inflammatory drugs).
